# arrayCGHbase: an analysis platform for comparative genomic hybridization microarrays

**DOI:** 10.1186/1471-2105-6-124

**Published:** 2005-05-23

**Authors:** Björn Menten, Filip Pattyn, Katleen De Preter, Piet Robbrecht, Evi Michels, Karen Buysse, Geert Mortier, Anne De Paepe, Steven van Vooren, Joris Vermeesch, Yves Moreau, Bart De Moor, Stefan Vermeulen, Frank Speleman, Jo Vandesompele

**Affiliations:** 1 Center for Medical Genetics, Ghent University Hospital, De Pintelaan 185, B-9000 Ghent, Belgium; 2 Department of Electrotechnical Engineering, Faculty of Applied Sciences, Katholieke Universiteit Leuven, Kasteelpark Arenberg 10, B-3001 Heverlee, Belgium; 3 Center for Human Genetics, Leuven University Hospital, Herestraat 49, B-3000 Leuven, Belgium; 4 Hogeschool Gent, Vesalius Department of Health Care, Keramiekstraat 80, B-9000 Ghent, Belgium

## Abstract

**Background:**

The availability of the human genome sequence as well as the large number of physically accessible oligonucleotides, cDNA, and BAC clones across the entire genome has triggered and accelerated the use of several platforms for analysis of DNA copy number changes, amongst others microarray comparative genomic hybridization (arrayCGH). One of the challenges inherent to this new technology is the management and analysis of large numbers of data points generated in each individual experiment.

**Results:**

We have developed *arrayCGHbase*, a comprehensive analysis platform for arrayCGH experiments consisting of a MIAME (Minimal Information About a Microarray Experiment) supportive database using MySQL underlying a data mining web tool, to store, analyze, interpret, compare, and visualize arrayCGH results in a uniform and user-friendly format. Following its flexible design, *arrayCGHbase *is compatible with all existing and forthcoming arrayCGH platforms. Data can be exported in a multitude of formats, including BED files to map copy number information on the genome using the Ensembl or UCSC genome browser.

**Conclusion:**

*ArrayCGHbase *is a web based and platform independent arrayCGH data analysis tool, that allows users to access the analysis suite through the internet or a local intranet after installation on a private server. *ArrayCGHbase *is available at .

## Background

The introduction of a microarray based comparative genomic hybridization method (arrayCGH) in 1997 paved the way for higher resolution detection of DNA copy number aberrations [1]. ArrayCGH is founded on the same principles as metaphase CGH, but uses mapped reporters instead of whole chromosomes. One of the major challenges in arrayCGH studies remains the accessibility, management, and interpretation of the vast amount of data generated in single experiments, and parallel comparison of multiple experiments. Typically, these arrays contain 3,000 to 30,000 reporters, each of which has multiple biological annotations (chromosomal position, sequence information, gene name, biological and molecular function,...) as well as physical (grid layout) and quality control (sequence verification, FISH mapping information,...) annotations. In addition, the description of the DNA samples under investigation and the applied lab protocols should be easily accessible. For classical CGH, several commercial software packages are available to analyze and interpret the data of a CGH experiment. Also for arrayCGH there are a number of separate software systems that individually address some of the needs, such as databases for data storage (BASE [2]), applications for clustering and visualization of microarray data (seeGH [3], M-CGH [4], CGHAnalyzer [5], aCGH-smooth [6] and CGH-Miner [7]), public genome databases that contain reporter information, commercially available Laboratory Information Management Systems (LIMS), and various storage methods for recording biomaterial annotations. However, none of these software packages or databases combine all these features (see Supplemental Table). In this paper, we present the development of a web based open source arrayCGH analysis platform, *arrayCGHbase*, that combines all these features and on top provides additional unique aspects making the analysis and sharing of arrayCGH data easily implementable for both research and routine purposes.

## Implementation

### MIAME compliant database

**Figure 1:**
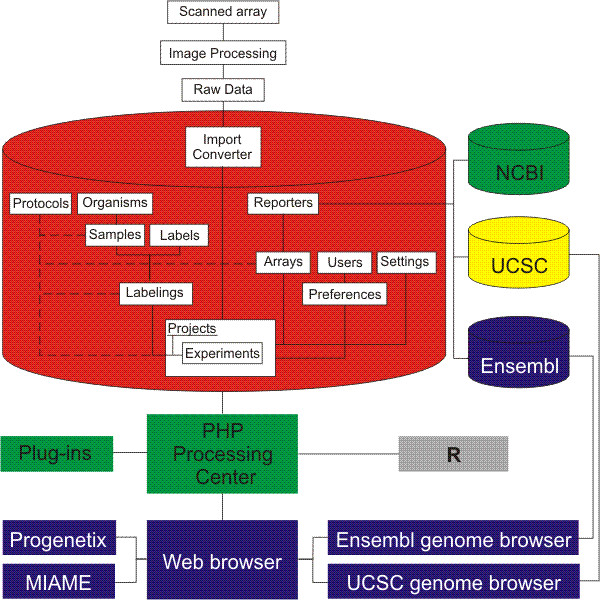
***arrayCGHbase *scheme**. The data is stored in a MIAME supportive MySQL database (red) and reporter info is updated using the NCBI, Ensembl and UCSC genome database. Data and results are presented to the user through a web browser via the PHP scripting language. Data-normalization and other analysis or result visualization methods can be integrated using the plug-in architecture. Further data processing using the R statistical scripting language will be implemented in the near future. Results can be exported to a Progenetix or MIAME compatible format, or visualized on the genome using the Ensemble or UCSC genome browser.

*arrayCGHbase *runs in Windows, Linux, Macintosh, and Unix environments. Particular attention was paid to the use of open source software for the development of *arrayCGHbase*. The software was developed in the PHP scripting language, with all data being stored in a relational, MIAME [8] (Minimal Information About a Microarray Experiment) supportive, MySQL database and communicated to the user through an Apache Web server (Figure 1). After installation on a private server, experiments can be shared by different users over the internet or a local intranet. *ArrayCGHbase *integrates DNA sample information, lab protocols, extracted data, and contains a plug-in architecture for data transformation, analysis, and graphical display, allowing users to develop their own modules. Reporters can be directly linked to the Ensembl [9] or UCSC [10] genome browsers, providing additional up-to-date information on each reporter. Reporters can also be manually imported into the MySQL database with the ability to update all linked experiments. The structure of *arrayCGHbase *was designed to follow the laboratory workflow and is compatible with all types of arrayCGH experiments and data formats (dual colour genomic clone, cDNA [11], or oligonucleotide [12] arrays spotted on any substrate, physical layout, type of array, as well as single channel hybridizations such as the Affymetrix SNP chips [13]). With a personal account and administrated access levels, a user can enter new DNA samples, annotate these, and append all relevant sample information such as quantity, quality, and applied lab protocols at each step. Each user can group experiments together into projects and, in a uniform and streamlined fashion, apply filters and transformations and run analyses. Data is exportable in several formats for offline analysis using other (dedicated) software tools, for publication or for sharing data with the research community. For advanced users, an SQL query window allows interrogation of the underlying MySQL database.

### Data processing and visualization routines

A first and important step in data analysis of arrayCGH experiments is the processing of large, possibly noisy data sets to identify the specific reporters that are differentially hybridized and hence show an aberrant copy number. Data processing is performed in a streamlined four-step manner: (1) the local noise or background associated with the experiments is removed, (2) the quality of the experiment is assessed and poor quality features are removed, (3) ratios are calculated, transformed to log_2 _scaled ratios, and normalized, and finally (4) reporters that show altered ratios are identified and hence, reporters with aberrant copy number are identified. In the past, this normally required the sequential processing of data by different, often incompatible programs. Using established and widely used microarray (CGH) data processing procedures, *arrayCGHbase *will automatically correct the signal intensities, filter out unwanted poor quality features (based on signal to noise ratio, image processing software related flags, or other user defined filters), normalize the fluorescence intensity ratios, score levels of differential hybridization, combine the results of replicate experiments and assess the quality of individual and replicate experiments. All these steps are user adjustable.

### Input data and local background correction

The experimental input data for *arrayCGHbase *consists of export files generated by image analysis software. Currently, the program recognizes files from GenePix Pro versions 2.0–4.0, Scanalyze version 2.0, UCSF SPOT version 2.0, Imagene versions 4.0 – 5.5 and the Affymetrix Chromosome Copy Number Tool. The program can easily be updated for the recognition of other data input formats upon request. Moreover, *arrayCGHbase *has an interactive import wizard, which makes it possible to import data at your own desire. The processing steps may be changed by altering the parameters at the input stage. By default, the results for each feature are defined as the median foreground minus background intensities for each dye (as determined by the image processing software). The ratio of each feature is determined as the relative background corrected signal between the two dyes or in the case of single color experiments as the corrected signal intensity.

### Poor quality flagging

Nearly every experiment contains features of poor quality, comprising features that have unusual morphology (e.g. doughnut patterns), exhibit uneven hybridization, or have saturated signal intensity. After background corrections, *arrayCGHbase *can automatically flag features of inferior quality using different criterions (e.g., the standard deviation between replicates), by a manually set signal or signal-to-noise threshold, or using image processing generated flag annotations.

### Normalization

Following calculation of the corrected signal intensities and filtering for good quality features, the relative contributions of the fluorescence intensities are compared. To go from a multiplicative space to an additive space, ratios are log_2 _transformed. Ideally, the signals of the two dyes should be equal for nucleic acid reporters that have equal amounts in the test and reference samples (i.e., the log_2 _transformed ratios of the two corrected signals should approach zero for reporters hybridizing to an equal degree in both fluorescence channels). However, in practice the ratio of the corrected signal intensities deviates from the expected ratio due to the different molecular and physical characteristics of the dyes, the different amounts of DNA used for labeling with the different dyes, the spatial heterogeneity in the hybridization conditions across the slide, and many other factors. Normalization compensates these effects by applying a data transformation such that ratios of reporters with unchanged copy-number are close to zero. In the normalization step, an appropriate term is added or subtracted from the log_2 _transformed ratio for each feature. The program allows normalization in several ways, either by global normalization or subgrid (or pin) normalization, or by a combination of different normalization procedures.

A major issue in microarray normalization is the definition of the set of constant probes to which the data are normalized. The most widely accepted method employs the 'constant majority' method, which assumes that the majority of reporters do not change in ratio. This method, which is implemented in *arrayCGHbase*, is generally applicable to most experiments as it is valid even in cases where up to 50% of reporters have altered ratios, it does not require prior knowledge of which features remain constant, and allows for intensity and spatial variation. Hence, this method calculates a scaling term from the median of all ratios, excluding all outliers. In this way the distribution of all ratios is transformed so that it centers around zero.

### Quality control

#### Percentage of good quality spots

This first quality assessment is a basic calculation of the number of reporters (or features) that are not flagged based on quality measures (user defined parameters and thresholds, see above).

#### Intra- and inter-array hybridization quality

Three other major quality parameters can be determined with *arrayCGHbase *for each experiment. The first assesses the variation between reporters present in replicates on the array (typically duplicates or triplicates). An increased variation typically reflects lower quality hybridizations resulting in less reliable ratios. A second quality parameter is the standard variation between the different reporters on the array that show a normal (unaltered) copy number. This quality measure is only applicable in experiments with few reporters with aberrant copy number. The third quality measure is the average ratio for reporters with aberrant copy number. This ratio should significantly differ from zero to allow identification of differentially hybridized reporters. This last quality measure is only applicable in experiments where DNA copy number aberrations are known or validated. These parameters provide an objective quality measure and can also be helpful to compare different experiments.

**Figure 2:**
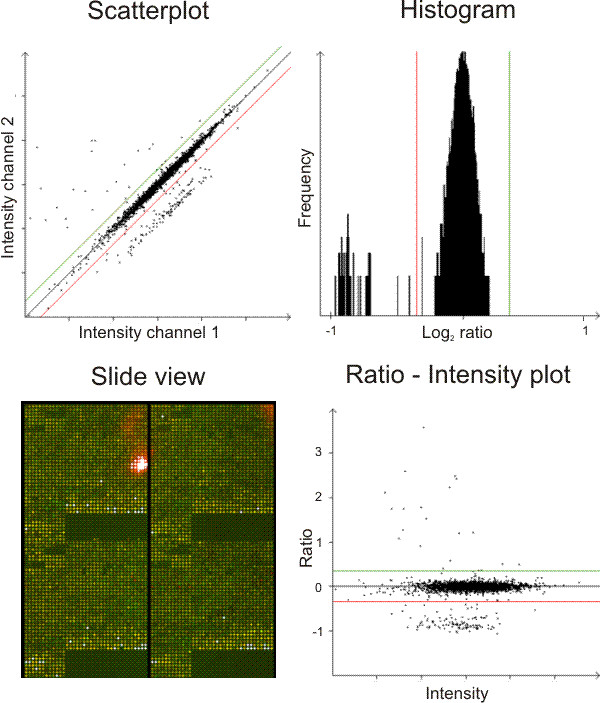
**Quality control graphs**. Graphical displays to assess the quality of an experiment, such as a dual channel intensity scatter plot, ratio histogram, ratio-intensity plot and a virtual slide view.

In addition to these parameters, different graphical displays, such as ratio-intensity plots (usually referred to as MA plots), dual channel intensity scatter plots, and ratio histograms give an idea of the quality of an individual experiment or series of experiments (Figure 2). In all these visualizations, thresholds for gains and losses are displayed and can be adjusted. The slide viewer generates a virtual spatial view of all features on the array using the ratio, or signal and background intensities; this viewer allows the identification of problematic regions or artifacts on the slide surface. Clicking on an individual feature shows specific data associated with this feature (e.g., reporter name, signal intensities, and data quality flags).

### Scoring chromosomal regions with aberrant copy number

The final step in arrayCGH data processing is the identification of reporters that exhibit differential hybridization, corresponding to chromosomal regions that have altered copy number. The major issue is to identify those reporters whose relative ratios stand out from the experimental noise with sufficient statistical significance. *arrayCGHbase *currently incorporates two scoring methods. The most widely used approach is to define a ratio threshold and identify the probes that exhibit ratios greater or smaller than this threshold. Another, statistically more sound approach, is to use a floating threshold based on the standard deviation of all reporters in a given experiment. Reporters that exhibit ratios greater than this threshold will be defined as differential [14]. Both methods are implemented in *arrayCGHbase *and can be applied on each individual feature, or on the mean value of replicates. Besides the aberrant feature scoring methods, two other algorithms are available: a universal data smoothing algorithm, as well as a breakpoint-identification algorithm, which both consist of a moving window along the chromosomes and hence make use of the spatial "along the chromosome" distribution of the reporters. With these algorithms, chromosomal breakpoints can be easily identified in more noisy datasets. By writing custom plug-ins (in PHP or R), sophisticated algorithms that use segmentation methods (e.g. Cluster Along Chromosomes, CLAC [7]) or others, can be implemented by any user in a straightforward way.

### Chromosome visualization

**Figure 3:**
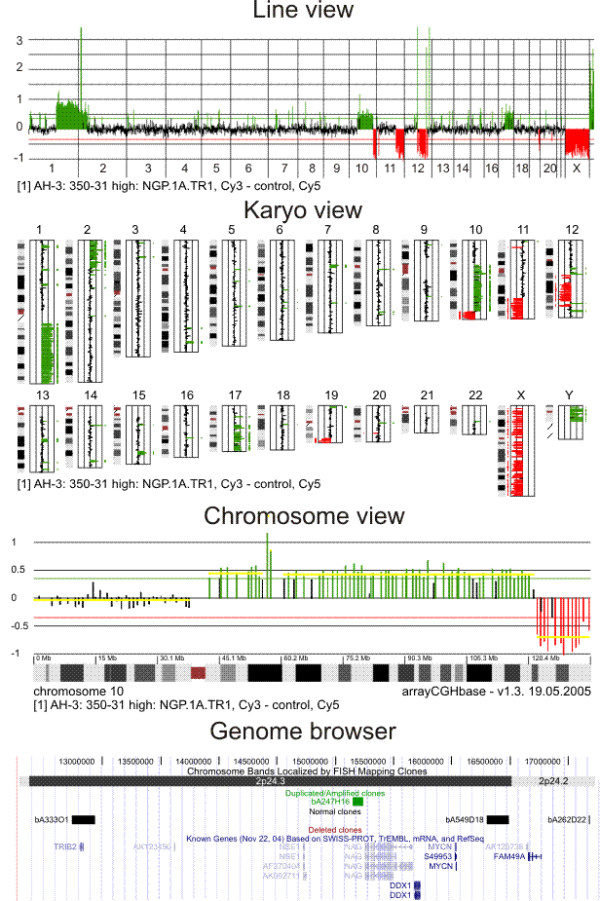
**Selected result viewers**. Graphical displays of *arrayCGH *results of neuroblastoma cell line NGP.1A.TR1: line view (all reporters ordered by chromosome and chromosomal position on one line), karyo view (al reporters mapped on their chromosomal position on a standard ISCN ideogram), chromosome view (zoom on one chromosome or chromosomal region) with breakpoint identification algorithm, and genome browser view (neuroblastoma cell line IMR32), with all reporters and their copy number status displayed in the UCSC genome browser.

A wide variety of result viewers are available. The results can be mapped upon standard ISCN (International Standard on Cytogenetic Nomenclature) ideograms in an electronic karyotype, or visualized per chromosome or zoomed in on a region of interest (Figure 3). Moreover, various CGH profile views provide the user with a tool to compare different experiments and to identify regions with relevant copy number alterations. Views are returned to the user either as PNG (Portable Network Graphic) or as SVG (Scalable Vector Graphic) files, with the ability to scale images according to screen width.

### Data export

Processed data can be exported as MIAME compliant text files and figures; these include the original feature signal and background intensities, the normalized ratio value, a list of reporters that are differentially hybridized, and the data quality parameters. Additionally, a file can be generated for submission of arrayCGH results directly into Progenetix [15], a comprehensive collection of published cytogenetic abnormalities in human neoplasms. Lastly, BED files can be created to map results and visualize the experiment from within the Ensembl or UCSC genome browser.

## ArrayCGHbase at work

In several publications from our research group, *arrayCGHbase *has been successfully used to analyse arrayCGH data to identify and delineate copy number aberrations [16-19]).

At the demo site, users can explore the data published in Hellemans et al. [16], a small ~5 Mb deletion in chromosome 12q identified using SNP chips), the results of a case report of the identification of an unbalanced X-autosome translocation by arrayCGH in a boy with a syndromic form of chondrodysplasia punctata brachytelephalangic type [17], a distal 9p trisomy and distal Xp nullisomy caused by an unbalanced X;9 translocation: 46, Y, der(X)t(X;9)(p22.32;p23) detected with a 1 Mb BAC array), and the copy number profile of a cancer cell line NGP.1A.TR [18]). It is possible to look at the raw data of these hybridizations and more importantly, test the performance of the program using different settings.

## Conclusion

We present *arrayCGHbase*, a versatile web based, platform independent data storage and analysis tool for processing microarray CGH data. Routines were implemented for feature flagging, data normalization, data quality assessment and the identification of chromosomal regions with aberrant copy number. A zoomable graphical interface allows immediate identification of altered genomic regions and the underlying gene content by several database links. A multitude of export functions allow the user to further process the results. The easy plug-in architecture makes it possible for each user to add custom algorithms for data analysis and visualization and share these with the user community. This webtool and database will enable investigators to interpret single experiments and compare large data sets efficiently throughout different array platforms and provides all of the essential features and links for further investigation of the genomic regions of interest.

## Future developments

*arrayCGHbase *will continually be updated to incorporate new processing methods that will be developed both within and outside our laboratory. Immediate plans include the addition of export and import functions to R [20] or Bioconductor [21] to be able to apply several available mathematical algorithms such as two-dimensional LOWESS normalization [22]. Immediate export functions to the DECIPHER web site [23] to link phenotypical data to actual experiments will also be included. The *arrayCGHbase *source code is freely available under a Creative Commons License, to encourage others to develop new analysis methods and utilities that will further improve its capabilities.

## Availability and requirements

An *arrayCGHbase *demo site is available at . At this site, all quality control features and other features can be tested for several experiments with BAC arrays as well as SNP chips (see '*arrayCGHbase *at work'). At the same site, the complete package can be freely downloaded for local installation on a private hosted web server. For local use, additional software is required such as the MySQL database [24], a web server (e.g. Apache [25]), and PHP hypertext preprocessor [26]. These software packages are freely available and are key parts of LAMP (Linux, Apache, MySQL, PHP), an open source web platform. Enquiries for *arrayCGHbase *should be made to arrayCGHbase@medgen.ugent.be.

## Glossary

Reporter: any DNA fragment (BAC, PAC, cosmid, fosmid, cDNA clone, oligonucleotide, genomic PCR product) used for hybridization

Feature: physical reporter spotted, printed, or otherwise linked to a substrate at a specific location

PHP: Hypertext PreProcessor (server-side scripting language)

MIAME: Minimal Information About a Microarray Experiment

MySQL: My Structured Query Language

ISCN: International System for human Cytogenetic Nomenclature

BED: Browser Extendable Data

## Authors' contributions

BM was the principle programmer of arrayCGHbase. FP, KDP, PR and SVV contributed ideas for different features and display requirements. JV oversaw the project; all other authors have reviewed the manuscript and FS and JV were the final editors of the manuscript.

## Supplementary Material

Additional File 1 Comparison between different already available arrayCGH software programs and arrayCGHbase for the analysis and visualization of arrayCGH data. Click here for file
